# Phenotypic variability and management of patients with mosaic monosomy X and Y chromosome material: a case series

**DOI:** 10.1186/s13052-024-01618-9

**Published:** 2024-05-07

**Authors:** Myriam Ben Fredj, Marwa Messaoud, Sabrine Ben Youssef, Salma Mani, Syrine Laaribi, Rania Sakka, Hayet Ben Hmida, Amine Ksiaa, Mongi Mekki, Mohsen Belghith, Lassaad Sahnoun

**Affiliations:** University of Monastir Faculty of Medicine of Monastir, Université de Monastir faculté de medicine de Monastir, Monastir, Tunisia

**Keywords:** 45X, 46XY; 45X, 46, X, Der(Y); mosaicism; phenotype; Monosomy, Disorder of sex development

## Abstract

**Background:**

we aim to discuss the origin and the differences of the phenotypic features and the management care of rare form of disorder of sex development due to Mosaic monosomy X and Y chromosome materiel.

**Methods:**

We report our experience with patients harboring mosaic monosomy X and Y chromosome material diagnosed by blood cells karyotypes and cared for in our department from 2005 to 2022.

**Results:**

We have included five infants in our study. The current average age was 8 years. In four cases, the diagnosis was still after born and it was at the age of 15 years in one case. Physical examination revealed a variable degree of virilization, ranging from a normal male phallus with unilateral ectopic gonad to ambiguous with a genital tubercle and bilateral not palpable gonads in four cases and normal female external genitalia in patient 5. Karyotype found 45, X/46, XY mosaicism in patient 1 and 2 and 45, X/46, X, der (Y) mosaicism in patient 3, 4 and 5. Three cases were assigned to male gender and two cases were assigned to female. After radiologic and histologic exploration, four patients had been explored by laparoscopy to perform gonadectomy in two cases and Mullerian derivative resection in the other. Urethroplasty was done in two cases of posterior hypospadias. Gender identity was concordant with the sex of assignment at birth in only 3 cases.

**Conclusion:**

Because of the phenotypic heterogeneity of this sexual disorders and the variability of its management care, then the decision should rely on a multidisciplinary team approach.

## Background

Mosaic monosomy X and Y chromosome material is characterized by the coexistence of two or more cell lines (e.g., 45, X/46, XY or 45,X/46,X, der(Y)) originating from mitotic nondisjunction in postzygotic embryonic development [1]. It is responsible for a disorder of sexual development, with a phenotype ranging from Turner syndrome to individuals with genital ambiguity to a normal male phenotype with spermatogenesis abnormalities [2]. This variability depends on two factors: the distribution of this mosaicism in organs and systems, and the structure of the Y chromosome [3].

Due to the constraints of cytogenetic study resources in our country and considering the socio-cultural conditions, these types of disorders of sexual development continue to be challenging in the gender assignment, particularly at birth.

The aim of this study is to discuss the origin and the differences of the phenotypic features and to highlight the difficulties of sex orientation in the baby with mosaic monosomy X and Y chromosome material.

## Materials and methods

The study was conducted retrospectively at the pediatric surgery Department. All cases with monosomy X with Y chromosome material managed at our department from 2005 to 2022 were enrolled in the study. Demographic data was collected from patient’s files included age of presentation, prenatal history, external genitalia features, sex of assignment at birth, dysmorphic features, hormonal profile, radiologic findings by abdominopelvic ultrasound and MRI, internal genitalia findings by laparoscopy or coelioscopy, gonadal histology features and expressed gender identity. Since tests, namely Comparative Genomic Hybridization using microarray technique (CGH) and karyotyping on gonadal cells, are not available at our center, chromosomal analysis was done only by a standard karyotype from the blood lymphocytes combined with SRY gene characterization by flurorescent in situ (FISH). All the cases were discussed during a multidisciplinary meeting to establish the management decision. Surgical management methods and outcomes were also recorded.

## Results

We included in our study five patients presenting with monosomy X with Y chromosome material confirmed by the karyotype. The age of patients at diagnosis varied from antenatal (case 2), at birth (3 cases) to the age of 13 years old in case 5.

### Clinical features

#### Case 1

On the systematic newborn examination, a curved penis with posterior hypospadias and peno-scrotal transposition was observed, accompanied by the absence of a gonad in the right scrotum and a palpable gonad on the left. The assigned gender at birth was male with a further complementary test. The genitography outlined a normal male urethra. The laparoscopic exploration identified a hypoplastic uterus, absence of gonad on the right, and a vas deferens with testicular vessels on the left.

#### Case 2

On the antenatal ultrasound, an intra-abdominal cystic formation was noted. A normal phallus and unilateral gonadal ectopia was observed on clinical examination. The internal genital organs were explored laparoscopically and found a vagina opening into a urogenital sinus, an uterus, a left fallopian tube, and hypoplastic spermatic vessels on the right. Genitography disclosed a 5 cm long urogenital sinus with opacification of an oblong median cavity. After karyotype the assigned gender was male.

#### Case 3

The masculin gender was assigned at birth. Clinically, he had a normal-sized phallus, scrotal hypospadias, a right gonad in the scrotum, and an inguinal gonad on the left, accompanied by a hypoplastic left scrotum and bilateral inguinal hernia. No Müllerian derivatives were identified. Genitography disclosed a normal male urethra.

#### Case 4

The assigned gender was female. She had a feminine phenotype with an internally normal female genital organs, including a uterus and a vagina opening into a common urogenital sinus measuring 2.5 cm detected by genitography. External genital examination revealed a genital tubercle with fused labioscrotal swellings. Pelvic MRI did not detect any intra-abdominal gonad.

#### Case 5

A 13 years old child was managed for primary amenorrhea. The clinical examination revealed: a short and webbed neck, a shield chest, pectus excavatum, a low nuchal hairline, widely spaced and hypoplastic nipples, a prepubertal Tanner stage, and normal female external genitalia with no palpable inguinal masses. Then Turner syndrome was suspected, leading to the request for a karyotype analysis, which revealed mosaicism 45, X/46, X,idic(Y).

Endocrine tests including plasma levels of 17-hydroxyprogesterone, testosterone, anti-Müllerian hormone (AMH), and cortisol were all normal in patients 1 to 4. At diagnosis, patient 5 had an ovarian insufficiency with low levels of estradiol and markedly high levels of LH and FSH. She also had a peripheral hypothyroidism.

Abdominopelvic ultrasound revealed no renal malformations in any of our patients. Cardiac ultrasound detected aortic coarctation in patient 5, who is currently under the care of a pediatric cardiologist.

### Cytogenetic diagnosis

All patients received a cytogenetic diagnosis postnatally. Patients 1 and 2 presented with 45, X/46, XY mosaicism and patients 3, 4 and 5 presented with 45, X/46, X, der (y) mosaicism. The rearranged Y chromosome was characterized as a ring chromosome in patient 3, an isochromosome isodicentric in patient 5 and was not identified in patient 4 because we could not characterize it. The Y chromosome carried SRY gene in all cases. In all patients, percentages of monosomic X cell line were nearly similar with predominating Y chromosome- harboring cell line at 60% except for patient 4 who had a predominating monosomy X cell line at 85%.

Following cytogenetic diagnosis revealing Y material, patients were assigned to the male gender except for patient 4 who had severe ambiguous undervirilized external genitalia and for patient 5 who was diagnosed in late infancy.

### Surgical management

A resection of the Müllerian derived structures was performed in two cases (1 and2). In the second case, the resection was associated to a gonadectomy due to the intimate adhesions of the right spermatic vessels and vas deferens to uterus. Furthermore, for case 3, an orchidopexia with a hernia repair was performed.

A masculinizing surgery was performed for case 1 and 3 to correct posterior hypospadias with severe chordee using Koyanagui urethroplasty at the age of 2.5 years and 3 years respectively. This surgery was preceded by hormone stimulation to increase the penile length.

For case 4, feminizing surgery was performed, including clitoridoplasty, vaginoplasty using a Fortunoff flap, and labioplasty.

Finally bilateral gonadectomy was recommended and performed for case 5 to prevent the risk of progression towards gonadoblastoma.

The histological examination without cytogenetic analysis showed ovarian stroma without differentiated gonadal structures in case 2 and both gonads made of fibroadipous streak gonad tissue and peripheral tubular structures resembling epididymis in case 5. There was no evidence of gonadoblastoma.

### Gender identity

We still ensure the follow-up of all our patients in collaboration among a pediatrician, geneticist, and pediatric surgeon for all our patients. Only case 1 has been lost to follow-up. During the follow-up and at the age of 8–9 years, the patients were referred to a pediatric psychiatrist for the assessment of their gender identity. Consequently, three of our patients, namely case 1 (17 years old), case 4 (8 years old) and case 5 (15 years old), exhibited gender identities consistent with their assigned sex at birth. The remaining two patients have not yet reached the age for the assessment of gender identity.

Table 1 summarizes the karyotypes formula, the phenotypic features and the management of the patients.

**Table 1:** Summary of phenotype and genotype findings in the reported cases

Cases	Patient 1	Patient 2	Patient 3	Patient 4	Patient 5
Family Medical History	− Unrelated parents − Testicular ectopia in two 46XY brothers	− Unrelated parents	− Unrelated parents	− Unrelated parents	− Unrelated parents
Prenatal History	− Born at full term − Vaginal delivery	− Born at full term − Vaginal delivery − History of urinary infection − Prenatal diagnosis of an abdominal cystic formation	− Born at full term − Vaginal delivery	− Born at full term − Vaginal delivery	− Born at full term − Vaginal delivery
Age at presentation	Shortly after birth	Prenatal period	Shortly after birth	Shortly after birth	13 years
Birth sex assignment	Male	Male	Male	Female	Female
External genitalia phenotype	− Penile curvature − Posterior hypospadias − Penoscrotal transposition	− Normal phallus − Unilateral ectopic gonad	− Normal phallus size − Posterior hypospadias − Scrotum-located urogenital meatus − Hypoplastic left scrotum − Bilateral inguinal hernia	− 2.5-centimeter curved genital tubercle − Unique urogenital orifice − Fused labioscrotal swellings	− Normal prepubertal female
Internal genitalia phenotype	− Normal left vas deferens and related spermatic vessels − No right vas deferens was found − Hypoplastic uterus with round ligament	− vagina that ends in the posterior urethra − left fallopian tube cystic vagina − Uterus structure − Hypoplastic right-sided vas deferens	− No Müllerian-derived internal genitalia	− Normal internal female genitalia, including and uterus and vagina	− Normal internal female genitalia, including an uterus and vagina
Genitography	− Normal masculinized urethra	− 5 cm urogenital sinus with oblong cavity in the median pelvic axis	− Normal masculinized urethra	− 2.5 cm urogenital sinus with vertical female urethra	Not done
Karyotype	45,X/46,XY mosaicism	45, X/46, XY mosaicism	45,X/46X,r(Y) mosaicism	45,X/46,X, der(Y) mosaicism	45X/46X,idic(Y) mosaicism
Gonads	Left: fully descended left testis	Left: streak gonad	− Left: inguinal left testis	Left: gonad not found by pelvic MRI	Left:Streak Gonad
	Right: not found	Right: Fully descended testis	− Right: Fully descended right testis	Right: gonad not found by pelvic MRI	Right:streak gonad
Management	− Removal of the hypoplastic uterine structure and its adnexa. − Testosterone hormone therapy to increase penile length. − Masculinizing surgery	− Resection of Müllerian-derived structures (vagina, uterus, fallopian tube, and left ovary) − The right vas deferens was also resected due to its adherence to the uterine corn	− Inguinal hernia surgery − Masculinizing surgery − scheduled for laparoscopic surgery with resection of the mullerian derivatives	− Feminizing surgery, including clitoral reduction, vaginoplasty, and labioplasty	− Removal of dysgenetic gonads − Hormonal replacement
Outcomes	Favorable	Favorable	Favorable	Favorable	Favorable
Follow-up	17 Years	4 years	3 years	8 years	2 years
Gender identity at the age of 8 years old	Boy	Not yet clearly expressed	Not yet clearly expressed	Girl	Girl

## Discussion

The Y chromosome is responsible for the male identity of sex through the genes it carries, including the sex-determining gene SRY and spermatogenesis genes. In the Mosaic monosomy X and Y chromosome material, the degree of male sexual differentiation depends on the percentage of cells with an intact XY genotype. Different degrees of gonadal tissue dysplasia, ranging from mild to severe dysplasia, are associated with this condition. The streak gonad is thought to result from a loss of the Y chromosome in the monosomic cell line for chromosome X [4, 5]. Moreover, expression of α-inhibin and AMH tissue is weak or absent in severe testicular dysplasia and absent in streaks [5]. Likely right gonadal and its fallopian tube agenesis observed in case 1, can be due to the resorption of a partially differentiated gonad that succeeded to secrete a sufficient amount of AMH to make regress the homolateral Müllerian duct but failed to secrete an appropriate amount of testosterone to make develop the Wolffian duct and to maintain proper development of a testis. Subsequently, the dysgenetic gonad of case 1 could have regressed and resorbed at stages of fetal development. With the evidence of the presence of the SRY gene on the rearranged Y chromosome in blood cells, both palpable testes have developed in case 3 while streak gonads have developed compatible with endocrine ovarian insufficiency in case 5. According to Layman et al., Structural Y chromosome anomalies, including ring and isodicentric Y chromosomes, have been associated with normal, dysgenetic or even ovo-testis gonads and despite the presence and apparent normalcy of the testicles, these children are infertile due to azoospermia or severe oligospermia [6]. In addition to the percentage of sex chromosome mosaicism, the degree of differentiation of testes depends on the dynamic mosaicism that could lead to the loss of SRY gene during mitotic divisons in cell lines within the gonads in development [7]. In the presence of a low dose of SRY, testicular differentiation is markedly altered [8], Like seen in case 5. Despite Variable degrees of dysgenesis can be found in biopsies of palpable testes harboring mosaic monosomy X with Y chromosome material We didn’t perform biopsy of palpable testes as they have had always a testicular component [9, 10].

The karyotype analysis conducted on gonadal cells emerges as a pivotal factor influencing sexual differentiation and gender orientation, irrespective of the mosaicism percentage observed in the blood. Consequently, the dominance of the 45, X cell line in a gonad directs its differentiation towards ovarian tissue, whereas the presence of testicles indicates the existence of a Y cell line with a sufficient SRY product. In line with this, the series of Gole and al., underscores that the percentage of the 45,X or Y cell lines in the blood lacks reliability as an indicator for the child’s phenotype [11].

This variability in phenotype is evident in case 1, 2, 3, and 5 within our series, despite a consistent 60% presence of the Y cell line and the presence of SRY in all four patients.

Furthermore, within the same patient, such as in the case of patient 3, the presence of both a testicle on one side and an ovary on the other illustrates the intricate nature of these developmental variations.

Additionally, the presence of Müllerian derivatives appears to correlate with the degree of testicular dysgenesis. No Müllerian derivatives were found in case 3 with bilateral testis development, while they persist but remain hypoplastic in cases 1 and 2 with unilateral dysgenesis. In cases 4 and 5, where gonads are likely absent and bilateral streak gonads are present, respectively, Müllerian derivatives are fully present.

This type of Disorder of Sex Development (DSD) is commonly associated with renal and cardiac malformations, underscoring the importance of routinely recommending cardiac and renal ultrasound examinations in these children for early detection of such malformations [6]. In our series, only one case of congenital heart disease was observed in case 5.

The presence of a Y chromosome in girls with Turner syndrome elevates the risk of gonadoblastoma and benign gonadal tumors, with an increased susceptibility to malignant transformation. Hence, early gonadectomy is recommended for these girls [1, 11, 12]. This justifies the gonadectomy performed on patient 5. For male, the management of gonads remains a topic of controversy. In the case of dysgenetic gonads and given the risk of degeneration, resection is the standard practice. Laymann et al. advocate a conservative approach to the testicles, involving regular monitoring through clinical examination, testicular ultrasound, as well as hormonal and tumor marker assessments [6, 13]. In our management strategy, we retain the testicle in its anatomical position and reposition the ectopic one, and we assure closely monitoring for the risk of degeneration.

The removal of Müllerian derivatives and masculinizing surgery were needed in patients assigned male and feminizing surgery was required to address female gender concordant genitalia. Case 5 is managed according to the Turner syndrome guidelines [13].

As Stockholm et al., the mortality rate is elevated in comparison to the general population, doubling in males and quadrupling in females. This highlights the importance of extended follow-up targeting all organs [14].

The main limitations of this study include the small number of patients with a mosaic monosomy X cell line and Y chromosome material, as well as the absence of karyotyping results for other tissues. This hinders our ability to establish chromosomal phenotypic correlations. Nonetheless, our series underscores the phenotypic heterogeneity within this group of disorders among patients. A uniformly common presentation of this disorder is uncommon. Additionally, the management approach varies, and decisions are made through a multidisciplinary team assessment of numerous phenotypic aspects.

Given the phenotypic polymorphism observed in patients with Mosaic monosomy X and Y chromosome material, the assignment of sex to newborns should not be determined solely based on karyotyping. It is imperative to extend the investigation to include imaging techniques (genitography and MRI), laparoscopy, endoscopy with gonadal biopsy, hormonal tests, and to make decisions after consultation with experts.

## Conclusion

Mosaic monosomy X and Y chromosome material is a group of disorders of sexual development (DSD) that can be identified shortly after birth due to variable degrees of masculinization of the external genitalia. Percentages of mosaicism and the nature of the rearranged Y chromosome do not predict genital phenotype. Given the limitations of resources available in our center, particularly the inability to perform the Comparative Genomic Hybridization (CGH) technique and karyotyping on gonadal cells, a multidisciplinary approach involving genetic, radiological, surgical, and histological explorations facilitates appropriate gender assignment and comprehensive management of this malformation.

## Data Availability

The datasets used and/or analysed during the current study are available from the corresponding author on reasonable request.

## Abbreviations

DSD: disorder of sex development
FISH: fluorescence in situ hybridization
SRY: sex-determining region Y gene
AMH: Anti-Mullerian Hormone
CGH: Comparative Genomic Hybridization
MRI: Magnetic Resonance Imaging

## References

[1] -Huhtaniemi I. Encyclopedia of endocrine diseases. Academic; 2018.

[2] -Guzewicz L, Howell S, Crerand CE, Umbaugh H, Nokoff NJ, Barker J (2021). Clinical phenotype and management of individuals with mosaic monosomy X with Y chromosome material stratified by genital phenotype. Am J Med Genet Part A.

[3] - Gole LA, Lim J, Crolla JA, Loke KY (2008). Gonadal mosaicism 45, X/46, X, psu dic (Y)(q11. 2) resulting in a Turner phenotype with mixed gonadal dysgenesis. Singapore Med J.

[4] -MacLaughlin DT, Donahoe PK (2004). Sex determination and differentiation. N Engl J Med.

[5] -Lepais L, Morel Y, Mouriquand P, Gorduza D, Plotton I, Collardeau-Frachon S (2016). A novel morphological approach to gonads in disorders of sex development. Mod Pathol.

[6] -Layman LC, Tho SP, Clark AD, Kulharya A, McDonough PG (2009). Phenotypic spectrum of 45, X/46, XY males with a ring Y chromosome and bilaterally descended testes. Fertil Steril.

[7] -Iourov IY, Vorsanova SG, Liehr T, Monakhov VV, Soloviev IV, Yurov YB (2008). Dynamic mosaicism manifesting as loss, gain and rearrangement of an isodicentric Y chromosome in a male child with growth retardation and abnormal external genitalia. Cytogenet Genome Res.

[8] -Álvarez-Nava F, Soto M, Martínez MC, Prieto M, Álvarez Z. FISH and PCR analyses in three patients with 45, X/46, X, idic (Y) karyotype: clinical and pathologic spectrum. Annales De génétique. Elsevier; 2003. pp. 443–8. 10.1016/s0003-3995(03)00016-9 14659779

[9] -Holcomb GW III, St JPMSD, Peter. Holcomb and Ashcraft’s Pediatric Surgery. Edinburgh London New York Oxford Philadelphia St Louis Sydney. 2020;211–89.

[10] -Chang HJ, Clark RD, Bachman H (1990). The phenotype of 45, X/46, XY mosaicism: an analysis of 92 prenatally diagnosed cases. Am J Hum Genet.

[11] -Matsumoto F, Matsuyama S, Matsui F, Yazawa K, Matsuoka K (2020). Variation of gonadal dysgenesis and tumor risk in patients with 45, X/46, XY mosaicism. Urology.

[12] -Dowlut-McElroy T, Vilchez DA, Taboada EM, Strickland JL (2019). Dysgerminoma in a 10-year old with 45X/46XY Turner syndrome mosaicism. J Pediatr Adolesc Gynecol.

[13] -Colindres JV, Axelrad M, McCullough L, Smith EO, Huang GO, Tu DD (2016). Evidence-based management of patients with 45, X/46, XY gonadal dysgenesis and male sex assignment: from infancy to Adulthood. Pediatr Endocrinol Reviews: PER.

[14] - Stochholm K, Holmgård C, Davis SM, Gravholt CH, Berglund A (2024). Incidence, prevalence, age at diagnosis, and mortality in individuals with 45, X/46, XY mosaicism: a population-based registry study. Genet Sci.

